# Clinicopathological Spectrum of Benign Skin Adnexal Tumors in the Pakistani Population: A Single-Center Study

**DOI:** 10.7759/cureus.35753

**Published:** 2023-03-04

**Authors:** Rashida Saleem, Anila Chughtai, Asma Zafar, Ghazi Zafar, Ujyara Maryam Lone, Akhtar Chughtai

**Affiliations:** 1 Histopathology, Chughtai Institute of Pathology, Lahore, PAK

**Keywords:** skin adnexal tumors, nevus sebacus of jadasshon, poroma, pilomatricoma, hidradenoma

## Abstract

Background

Skin adnexal tumors (SATs) are categorized per the site of origin, for example, hair follicles, sebaceous glands, and sweat glands. In our population, there is limited information related to the clinicopathological characteristics of these tumors. Management and prognosis depend largely upon the morphological type of the tumor. In this study, we assessed the disease spectrum and most prevalent subtypes of benign SATs.

Methodology

An analysis of 565 cases was conducted in this cross-sectional study between January 2018 and December 2022, using a non-probability consecutive sampling approach. Patient age, gender, site of involvement, and diagnosis were documented according to the fourth edition of the WHO Classification of Skin Tumors published in 2018. Data were entered and analyzed using SPSS Version 26.0 (IBM Corp., Armonk, NY).

Results

Our study had 565 patients, out of which 271 (47.9%) were males and 294 (52.1%) were females. The mean age was 40.97±19.3 years (range, 2-100 years). Anatomical site variations were as follows, head and neck (n=336, 59.4%), extremities (n=124, 22%), trunk (n=84, 14.9%), and genital areas (n=21, 3.7%). The most common histological subtypes of benign SATs were sweat gland origin (n=350, 62.0%), followed by hair follicle origin (n=161, 28.5 %), and sebaceous gland origin (n=54, 9.5%).

Conclusion

Sweat gland tumors were the most prevalent class of benign SATs in our study, in which hidradenoma and poroma were the most frequent subtypes. Hair follicle origin was the second most prevalent class of tumors with pilomatricoma being the most frequent. Sebaceous tumors were overall uncommon; nevus sebaceous of Jadasshon was the most common tumor in this class.

## Introduction

Skin adnexal tumors (SATs) are a diverse group of tumors that arise from the skin adnexa, including hair follicles, sebaceous glands, and sweat glands. SATs are relatively uncommon, accounting for less than 5% of all skin tumors [1]. These tumors have a similar clinical presentation, as cutaneous swellings make them difficult to diagnose solely on a clinical basis. Hence, histological evaluation is mandatory for an exact diagnosis [1]. The biological nature of these tumors is the most important prognostic factor that determines the therapeutic approach. Hence, it is vital to establish the benign or malignant nature of these tumors and the exact histological subtype as per the classification of the World Health Organization [2,3]. Moreover, some of the subtypes are particularly associated with genetic syndromes and may be the first clinical presentation suggesting underlying syndromes, making the exact diagnosis/subtype crucial [4]. Some benign SATs have a tendency to transform into malignant counterparts over a period of time if left untreated; therefore, timely diagnosis and treatment are critical for patient management [5]. Some of the syndromes associated with SATs include *Birt-Hogg-Dube syndrome*, *Brooke-Spiegler syndrome*, *Cowden syndrome*, and *Muir-Torre syndrome* [6]. The purpose of this study is to assess the disease spectrum and most prevalent subtypes of benign SATs.

## Materials and methods

Study design

After approval from the institutional review board (CIP/IRB/1124), a cross-sectional study was conducted between January 2018 and December 2022 at one of the largest private pathology centers in the country, Chughtai Institute of Pathology, using non-probability consecutive sampling. All cases of benign SATs were histologically evaluated by three histopathologists and data were recorded on a proforma.

Inclusion criteria

All cases of benign SATs diagnosed between the above-mentioned duration regardless of gender, age, and procedure were included in the study.

Exclusion criteria

Biopsies with poor preservation, incomplete clinical information, unclassifiable tumors, and malignant SATs were excluded from the study.

Data collection

All cases of benign SATs were histologically evaluated by three histopathologists, and data were recorded regarding patient age, gender, site of lesion, number of lesions, gross appearance, and histological subtype on the proforma.

Statistical analysis

Data were analyzed using SPSS version 26.0 (IBM Corp., Armonk, NY, USA). Qualitative variables were expressed as percentages and frequencies. Quantitative variables were presented by mean and standard deviations.

## Results

**Figure 1:**
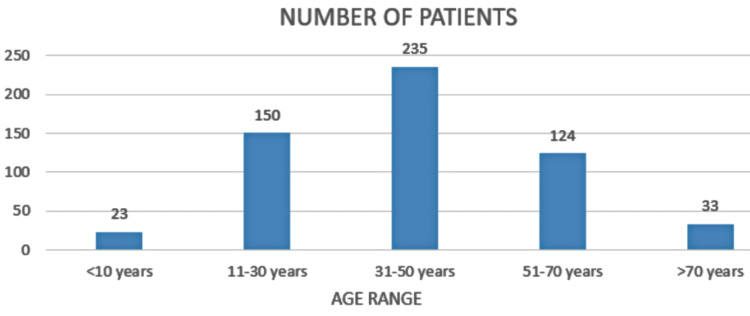
Number of patients among different age groups

Among the 565 included cases, 271 were males (47.9%) and 294 were females (52.1%). In this study, the mean age was 40.97±19.3 years (range 2-100 years). The mean age of females was 39.65 years (range 2-108) and for males, it was 42.33 years (range 3-90 years). Figure 1 shows the distribution of patients in different age groups.

In this study, 489 (86.5%) patients had a solitary lesion while 76 (13.5%) had multiple lesions. Benign SATs may appear as cystic nodules (n=409, 72.4%), solid nodules (n=116, 20.5%), or mixed solid and cystic nodules (n=40, 7.1%). There were anatomical site variations in the head and neck (n=336, 59.4%), extremities (n=124, 22%), trunk (n=84, 14.9%), and genital areas (n=21, 3.7%). It was reported that tumors of sweat gland origin made up the majority of benign SATs (n=350, 62.0%), of which hidradenoma (n=88, 15.5%) was most frequent. The second most common class of benign SATs was of hair follicular origin (n=161, 28.5%). Among them, pilomatricoma (n=83, 14.6%) was the most frequent. Tumors with sebaceous differentiation are overall uncommon (n=54, 9.5%) and nevus sebaceous of Jadassohn was the most common tumor in this class (n=29, 5.1%). Overall, the least prevalent benign SATs noted in our study were papillary eccrine adenoma (n=1, 0.1%) and dilated pore of Winer (n=1, 0.1%). The frequency of various benign SATs on the basis of their origin is shown in Tables 1-3.

**Table 1:** Frequency of different subtypes of benign SATs of sweat gland origin SATs: Skin adnexal tumors

Types of SATs	Frequency	Percentage
Hidradenoma	88	15.5%
Poroma	75	13.3%
Chondroid syringoma	48	8.5%
Spiradenoma	46	8.1%
Syringocystadenoma papilliferum	29	5.1%
Hidrocystoma	28	4.9%
Hidradenoma papilliferun	21	3.7%
Cylindroma	8	1.4 %
Syringofibroadenoma	4	0.7%
Syringoma	2	0.3%
Papillary eccrine adenoma	1	0.1%

**Table 2:** Frequency of different subtypes of benign SATs of hair follicle origin SATs: Skin adnexal tumors

SATs	Frequency	Percentage
Pilomatricoma	83	14.6%
Proliferating pilar tumor	28	4.9%
Inverted follicular keratosis	12	2.1%
Trichoepithelioma	12	2.1%
Trichofolliculoma	9	1.5%
Trichoadenoma	4	0.7%
Folliculosebaceous cystic hamartoma	4	0.7%
Trichoblastoma	2	0.3%
Pilar sheath acanthoma	2	0.3%
Fibrofolliculoma	2	0.3%
Trichilemomma	2	0.3%
Dilated pore of Winer	1	0.1%

**Table 3:** Frequency of different subtypes of benign SATs of sebaceous gland origin SATs: Skin adnexal tumors

SATs	Frequency	Percentage
Nevus sebaceus of Jadassohn	29	5.1%
Sebaceoma	15	2.6%
Sebaceous adenoma	10	1.8%

## Discussion

SATs are relatively uncommon cutaneous neoplasms. In routine practice, general pathologists encounter some of the subtypes frequently while others are infrequently encountered. Because of the rarity of some of the benign SATs, general surgical pathologists are unfamiliar with these, hence such uncommon entities pose diagnostic difficulties and are underreported. There have not been many detailed epidemiological studies focused on benign SATs. According to available data, there is significant variation in the overall prevalence of benign SATs across different regions. According to Hesari et al., 3.3% of Iranian biopsy series contained SATs [5,6]. However, Gonalez L et al. found a 1.4% prevalence of SATs in their study [7]. Benign SATs are more common than malignant SATs worldwide [8].

Since most of these tumors clinically present as skin swellings, histological evaluation is essential for accurate subtyping and assessment of biological behavior [1]. Understanding the biological behavior of these tumors is crucial for determining their prognosis [2,3]. Additionally, certain subtypes may develop malignant characteristics if they are not treated timely [5]. The purpose of this study was to evaluate the clinicopathological characteristics of benign SATs in our population. The mean age range was 40.97±19.3 years in our study, which is similar to the study conducted by Yaqoob N et al. [9], while the mean age was 28.2±9.45 years in a study published by Preeti et al., which is discordant with our findings [10]. Female gender predilection was noted in our study which is similar to the study conducted by Sam et al. [11]. This is in contrast to the study done by Bansal A et al. who cited male predominance [12]. We noted the most common site of involvement as the head and neck followed by the extremities and trunk. This is consistent with the findings of Subrata, Kaur, and Saman F et al. [13-15] and consistent with the study carried out by Thamilselvi et al. who reported extremities as the most common site [16]. Sweat gland tumors were the most common class of benign SATs noted in our study while Suri J et al. reported hair follicle tumors as the most common class [17]. Subrata et al. reported that hidradenoma is the most common of all benign SATs, which is in agreement with our study [13]. While findings of two different studies conducted in Turkey and Iran stated pilomatricoma as the most common subtype [6,8].

**Figure 2:**
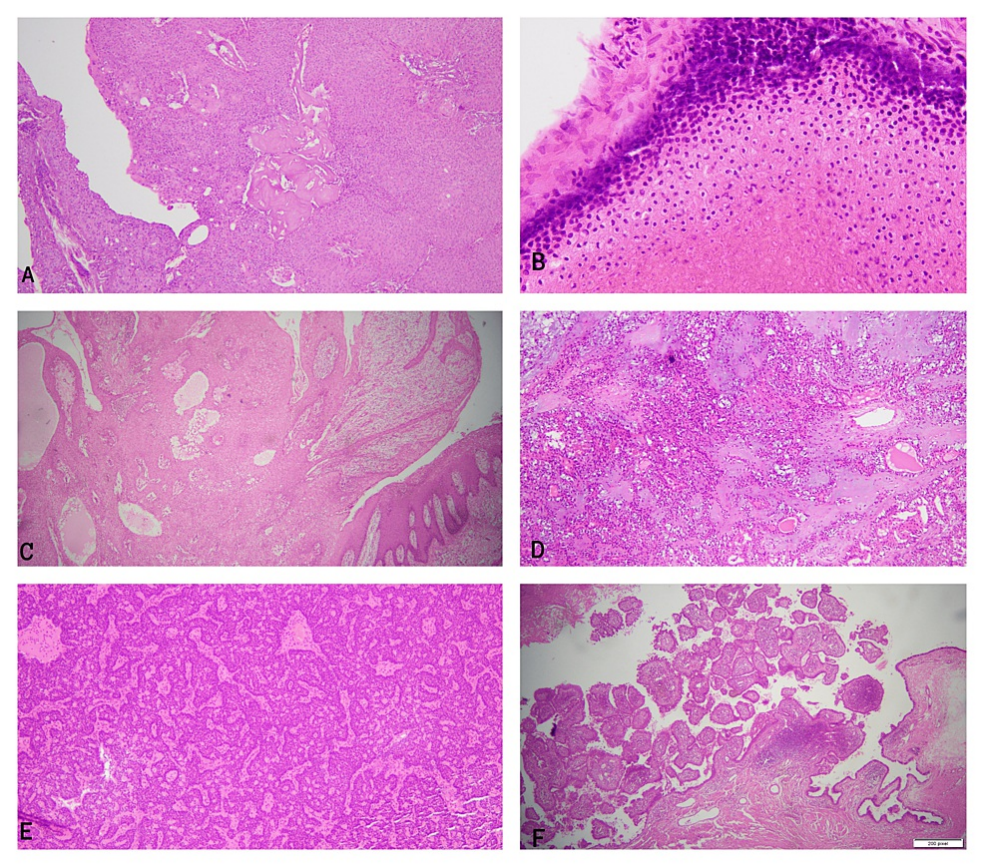
Microscopic pictures of different benign SATs A. Hidradenoma: Dermal-based lesion with eosinophilic to clear bland epithelial cells B. Pilomatricoma: Dermal-based lesion with round blue cells and ghost cells C. Poroma: Lesion with a broad spectrum with epidermis and showing small poroid cells D. Chondroid syringoma: Lesion showing a mixture of the epithelial and myoepithelial cells along with chondroid stroma E. Spiradenoma: Lesion with small blue cells with scattered reactive lymphocytes F. Syringocytadenoma papilliferum: Lesion with prominent papillary structures lined by bland sweat duct epithelium and plasma cells in the stroma SATs: Skin adnexal tumors

The three most common tumors reported in our study were hidradenoma followed by pilomatricoma and poroma. Histopathologically, hidradenomas have multilobulated solid and cystic architecture bland epithelial cells with clear eosinophilic cytoplasm and ductal differentiation [18]. Pilomatricoma is a dermal-based lesion characterized by anucleate pink 'ghost'/shadow' cells and small round blue cells representing germinative epithelial components [18]. Poromas have a broad surface epidermal connection and proliferate into the dermis as broad sheets of small round poroid cells. The presence of cuticle-lined ducts is an essential diagnostic feature [18]. Melanocytic matricoma, trichodiscoma, tubular adenoma, and tumor of follicular infundibulum were not reported during our study duration. Microscopic pictures of different benign SATs are shown in Figure 2.

Limitations

This was a single-center study with a limited sample size. No collaboration with local central registries can be established, further restricting the prevalence data. Larger population-based studies are recommended. Because of a lack of relevant clinical information, any association with genetic syndromes cannot be documented in this study. Moreover, the prognosis is not assessed in detail because of limited clinical input. Despite these limitations, the study provides valuable clinical and pathological information regarding benign SATs in the Pakistani population.

## Conclusions

Each subtype of benign SATs has characteristic morphological features that aid in the correct diagnosis. A lack of knowledge and expertise in general surgical pathologists due to limited exposure may lead to misclassification and underdiagnosis, ultimately affecting patient management. The main findings in our study included a wide age range, head-neck as the most common site, female gender predilection, and sweat gland tumors as the most common class followed by hair follicle tumors and sebaceous tumors, respectively. Hidradenoma and poroma were the most frequently encountered sweat gland tumors, Pilomatricoma and proliferating pilar tumors were the most common hair follicle tumors while nevus sebaceous of Jadassohn was most frequent in a sebaceous class of tumors. Melanocytic matricoma, trichodiscoma, tubular adenoma, and tumor of follicular infundibulum were not reported during our study duration. A larger sample size should be conducted in Pakistani populations in future studies.
